# Super-Resolution Ultrasound Imaging Can Quantify Alterations in Microbubble Velocities in the Renal Vasculature of Rats

**DOI:** 10.3390/diagnostics12051111

**Published:** 2022-04-28

**Authors:** Sofie Bech Andersen, Iman Taghavi, Stinne Byrholdt Søgaard, Carlos Armando Villagómez Hoyos, Michael Bachmann Nielsen, Jørgen Arendt Jensen, Charlotte Mehlin Sørensen

**Affiliations:** 1 Department of Biomedical Sciences, University of Copenhagen, 2200 Copenhagen, Denmark; stinnebyrholdt@gmail.com (S.B.S.); cmehlin@sund.ku.dk (C.M.S.); 2 Department of Diagnostic Radiology, University Hospital Rigshospitalet, 2100 Copenhagen, Denmark; mbn@dadlnet.dk; 3 Center for Fast Ultrasound Imaging, Department of Health Technology, Technical University of Denmark, 2800 Lyngby, Denmark; imat@dtu.dk (I.T.); jaje@dtu.dk (J.A.J.); 4 BK Medical ApS, 2730 Herlev, Denmark; carlos.villagomezhoyos@gmail.com; 5 Department of Clinical Medicine, University of Copenhagen, 2200 Copenhagen, Denmark

**Keywords:** contrast-enhanced ultrasound, ultrasound localization microscopy, kidneys, microvascular flow, Sprague Dawley rats, prazosin

## Abstract

Super-resolution ultrasound imaging, based on the localization and tracking of single intravascular microbubbles, makes it possible to map vessels below 100 µm. Microbubble velocities can be estimated as a surrogate for blood velocity, but their clinical potential is unclear. We investigated if a decrease in microbubble velocity in the arterial and venous beds of the renal cortex, outer medulla, and inner medulla was detectable after intravenous administration of the α1-adrenoceptor antagonist prazosin. The left kidneys of seven rats were scanned with super-resolution ultrasound for 10 min before, during, and after prazosin administration using a bk5000 ultrasound scanner and hockey-stick probe. The super-resolution images were manually segmented, separating cortex, outer medulla, and inner medulla. Microbubble tracks from arteries/arterioles were separated from vein/venule tracks using the arterial blood flow direction. The mean microbubble velocities from each scan were compared. This showed a significant prazosin-induced velocity decrease only in the cortical arteries/arterioles (from 1.59 ± 0.38 to 1.14 ± 0.31 to 1.18 ± 0.33 mm/s, *p* = 0.013) and outer medulla descending vasa recta (from 0.70 ± 0.05 to 0.66 ± 0.04 to 0.69 ± 0.06 mm/s, *p* = 0.026). Conclusively, super-resolution ultrasound imaging makes it possible to detect and differentiate microbubble velocity responses to prazosin simultaneously in the renal cortical and medullary vascular beds.

## 1. Introduction

Super-resolution ultrasound (SRUS) imaging allows mapping of the vasculature below the diffraction limit of conventional ultrasound, making in vivo microvascular ultrasound imaging possible [1]. An approach often used for SRUS is localizing and tracking individual intravascular microbubbles (MBs) over multiple successive image frames: a technique also called ultrasound localization microscopy [1,2,3]. The MBs have a small size (SonoVue mean size is around 2.5 μm), a viscosity similar to blood, and their rheology has been found comparable to that of erythrocytes [4,5,6,7,8]. Therefore, MB velocity can be used as a surrogate for blood velocity. Many strategies have been undertaken to link individual MBs across frames to generate reliable velocity estimations. Initially, MBs were tracked by connecting an individual MB to the nearest MB in the next frame or cross-correlating each MB’s intensity in small search windows between successive frames [2,3]. Since then, more advanced approaches have been proposed [9,10,11,12,13,14].

The unique arrangement of the renal vasculature is central in the filtration of plasma and the secretion/reabsorption processes that determine the final urine composition, all required for normal renal function. Additionally, renal blood flow alterations have been linked with, e.g., diabetic nephropathy and renal ischemia-reperfusion injury [15,16,17,18,19]. However, measuring the intrarenal blood flow is difficult due to the lack of in-depth microvascular imaging methods. From 2D SRUS data, estimations of the in-plane MB velocities in an entire image cross-section can be made. Accordingly, SRUS can evaluate renal cortical and medullary blood velocities simultaneously.

SRUS data have been used to study structural vascular alterations in different diseases, including chronic kidney disease, cancer, and vasa vasorum in atherosclerosis [20,21,22,23,24]. MB velocities have been extracted from healthy animal and human organs, including the kidneys [25,26]. However, studies showing that MB velocities can be used to quantify alterations in the vascular flow are sparse. In the cerebral vasculature of old mice, MB velocities were shown to decrease compared with younger animals [27]. In the eye of a rabbit, the MB velocities in retinal and retrobulbar vessels were shown to decrease with increasing ocular pressure [28], and recently, increased MB velocities were shown in renal cortical radial arteries of hypertensive rats compared with normotensive ones [29].

The MB velocity vectors are registered in velocity maps that show the MB tracks from all types of vessels in the image cross-section. In the mouse cerebral vasculature, the MB velocities were estimated across all vessel types in different functional brain areas [27] and, in the hypertensive rats, only velocities in the cortical radial arteries and veins were estimated, while the medullary microvasculature was not imaged [29]. Like the brain, the different anatomical areas of the kidneys have unique functions with a corresponding unique vascular anatomy and blood flow. This study aimed at investigating the quantitative possibilities of SRUS-derived MB velocities from the renal cortex and medulla of rats using a setup with a clinical ultrasound scanner and a hierarchical Kalman tracker for MB velocity estimation [10]. Prazosin is an α1-adrenoceptor antagonist that can substantially decrease renal blood flow via systemic vasodilation. We investigated if a decrease in MB velocity in the separated arterial and venous beds of the renal cortex, outer medulla, and inner medulla was detectable after intravenous prazosin administration.

## 2. Materials and Methods

### 2.1. Ethical Considerations

All procedures presented in this paper followed protocols approved by the National Animal Experiments Inspectorate under the Ministry of Food, Agriculture and Fisheries of Denmark (license number 2020-15-0201-00547 issued on 4 June 2020), project number P20-457 (28 August 2020). The experiments were ethically in accordance with the EU Directive 2010/63/EU for animal experiments. The rats were held in a 12/12-h light/dark cycle and could freely access standard chow and water. Trained animal caretakers were responsible for the rats’ well-being until the experiments.

### 2.2. Animal Preparations

Eight male *Sprague Dawley* rats were scanned; seven were included in the results (see exclusion criteria below). Physiological data on the rats are found in Supplementary Table S1. Initial anaesthetization was performed in a chamber supplied with 5% isoflurane (Vetflurane, 1000 mg/g, Virbac, Carros, France) in 65% nitrogen/35% oxygen, followed by tracheotomy, tube insertion, and ventilation with a 7025 Rodent Ventilator (Ugo Basile, Gemonio, Italy; 69 breaths/min). Subsequently, 1–2% isoflurane maintained the anesthesia. Two catheters were inserted in the left jugular vein: one for infusion of the muscle relaxant, Nimbex (0.85 mg/mL, GlaxoSmithKline, London, UK, 20 µL/min) and injection of prazosin hydrochloride (0.1 mg/kg, Sigma-Aldrich, St. Louis, MO, USA), and one for infusion of SonoVue (Bracco, Milan, Italy). A catheter in the right carotid artery was connected to a Gould Statham P23-dB pressure transducer (Gould, CA, USA), continuously recording the mean arterial pressure (MAP). The rats lay on a heating table (~37 °C) and laparotomy exposed the left kidney. A metal retractor on the left side of the diaphragm reduced respiratory motion transferred to the kidney.

### 2.3. Ultrasound Scanning and Prazosin Injection Procedure

The rats were scanned directly on the left kidney using a commercial bk5000 ultrasound scanner (BK Medical, Herlev, Denmark) modified for long data acquisitions. An X18L5s hockey-stick probe (BK Medical, Herlev, Denmark) was secured with a holder on the lateral side of the kidney to obtain central coronal image slices that included both the renal cortex and medulla. The image plane was found using orientation with B-mode. The SRUS data were obtained with line-per-line imaging (center freq. 10 MHz, mechanical index 0.1, frame rate 54 Hz) using amplitude modulation interleaved with B-mode imaging, the latter used for motion estimation (Supplementary Figure S1). The rats were scanned for 10 min using a 1:20 dilution of SonoVue infused at 40–55 µL/min (SP210iw syringe pump, WPI, Friedberg, Germany). Infusion rate was adjusted according to visible MBs on the contrast-enhancing scanner display. For continuous MB inflow, the syringe rotated 180° every 10 s. The MB concentration, center frequency, mechanical index, and acquisition time were adjusted in pre-trials to optimize SRUS imaging of both renal cortex and medulla.

**Figure 1:**
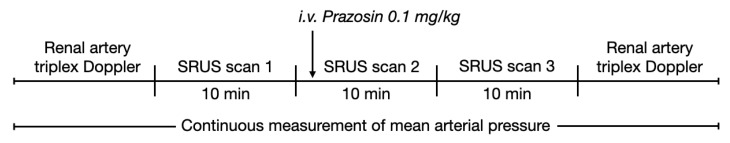
Timing of ultrasound scans and prazosin injection. Triplex Doppler included B-mode imaging, color Doppler, and spectral Doppler. i.v. = intravenous, SRUS = super-resolution ultrasound.

The rats were SRUS scanned three times (Figure 1). Prazosin was administered during the first minutes of SRUS scan 2. Triplex Doppler was acquired before SRUS scan 1 and after SRUS scan 3 on the renal artery or one of its branches. Rats were excluded if MAP < 70 mmHg at baseline or if there was no MAP response to prazosin. After the experiment, the rats were euthanized in anesthesia.

### 2.4. Super-Resolution Ultrasound Imaging Post-Processing and Region Labeling

The SRUS image processing pipeline is outlined in Supplementary Figure S1 and explained briefly here. In each B-mode frame, non-rigid motion was estimated in 3 × 3 mm patches with an 80% overlap [30]. In the contrast data, the MBs were detected after applying a threshold and Gaussian filtering, and their centroid localized using the weighted centroid algorithm. The MBs were then motion-compensated using the estimated motions and tracked with a hierarchical Kalman tracker [10]. Lastly, the generated MB tracks were inserted in high-resolution images to generate the SRUS images. By scaling and mapping the vector velocity information from the MB tracking to a multicolored (RGB) wheel, MB velocity maps that display the MB direction (color) and velocity (intensity, brighter colors are faster) were generated.

In MATLAB (R2020b, MathWorks, Natick, MA, USA), the MB velocity maps were manually labeled by S.B.A. with the three separate areas: cortex, outer medulla, and inner medulla (see example in the Results section). The cortex was delineated with an inner boundary (toward the medulla) superficial to the larger arcuate vessels that run between cortex and medulla. The outer medullas superficial boundary was set ~0.5–1 mm from the arcuate or segmental vessels, meaning that the outer stripe of outer medulla was not fully included. The loss of vascular bundles defined the transition from outer to inner medulla. In each area, smaller regions of interest were drawn in which a mean flow angle for the arterial flow was defined. A span of vessels going 125° (cortex) or 90° (medulla) from the mean angle was included as artery/arteriole tracks. As the afferent arterioles radiate at different angles from the cortical radial arteries, the vessel span was wider in the cortex. Vein/venule tracks were defined as tracks going in the opposite direction from the mean artery flow angle [31].

### 2.5. Microbubble Velocity Estimations

**Figure 2:**
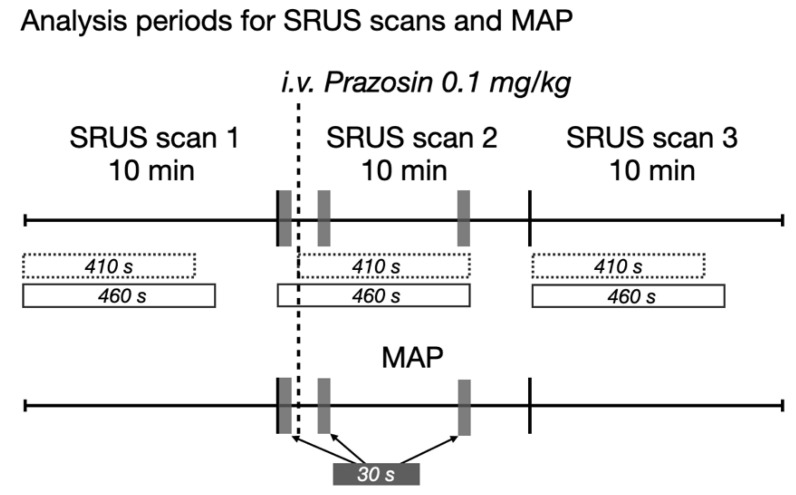
Overview of the analysis periods for the SRUS and MAP data. The 30-s periods compared in SRUS scan 2 and during MAP measurement are marked with grey blocks. The 410 s of SRUS data used to compare the mean microbubble velocity in the three scans are marked by the dotted-line blocks. The 460-s periods shown in the graphs with moving average microbubble velocities are the solid-line blocks. MAP = mean arterial pressure.

MB velocities were estimated in six different vascular beds: cortical arteries/arterioles, cortical veins/venules, outer medulla descending vasa recta, outer medulla ascending vasa recta, inner medulla descending vasa recta, and inner medulla ascending vasa recta. Generally, SRUS data must be acquired over a certain period depending on ultrasound equipment, acquisition technique, and vascular bed to obtain enough MB detections to generate a complete (or near-complete) image of the vasculature [32,33,34,35]. The MB velocities are typically calculated from the entire scan period to obtain the most reliable estimates; hence, the mean velocities from the three consecutive SRUS scans were compared. However, during a minute-long period, dynamic alterations in the blood flow can occur [36,37,38]. To better visualize dynamic alterations during scanning, the estimated MB velocities were displayed in graphs as moving averages (30-s window). The MB velocities from the following shorter periods of SRUS scan 2 were also compared to investigate the possibilities of quantifying the immediate response to prazosin: baseline velocity (first 30 s), the velocity at the max effect of prazosin on MAP (30 s with lowest MAP), and recovery velocity (last 30 s). Time for prazosin administration during SRUS scan 2 varied between animals from 51 to 184 s after scan start. Therefore, MAP and MB velocities from SRUS scan 2 were aligned according to the prazosin injection time, with moving average MB velocities starting from 50 s before injection and ending 410 s after injection for all rats. The mean MB velocity for SRUS scan 2 was estimated after prazosin injection resulting in 410 s of data. For SRUS scans 1 and 3, the first 410 s of the acquisition were used to match SRUS scan 2. An overview of the different periods is shown in Figure 2.

### 2.6. Statistical Analyses

Two variables (e.g., MB velocity in arteries/arterioles vs. veins/venules) were compared with a paired *t*-test. Three consecutive measurements (e.g., MB velocities from the three SRUS scans) were compared with repeated measures one-way ANOVA with a Greenhouse Geisser correction. Post-hoc between-group comparisons were made with Tukey’s multiple comparisons test. The scatter plots and graphs all show the means from the raw data. Statistical tests for the MB velocities were calculated on transformed data as they had a non-normal distribution (log- and square-root-transformed velocities in the cortex and medulla, respectively). Statistical tests, graphs, and plots were made in GraphPad Prism (version 9.2.0 for Mac, GraphPad Software, San Diego, CA, USA).

## 3. Results

### 3.1. Segmentation and Analysis of the Normal Renal Vasculature

**Figure 3:**
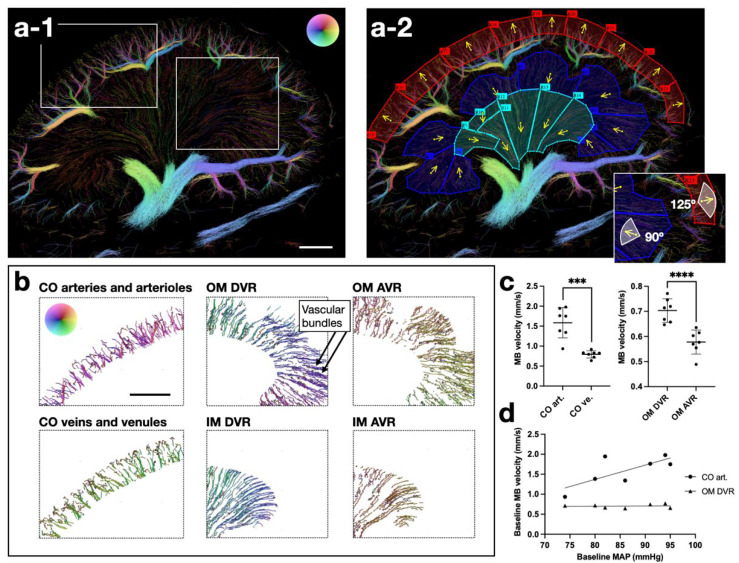
Segmentation of rat kidneys with separation of arteries/arterioles and veins/venules in the cortex, outer medulla, and inner medulla. (**a-1**,**a-2**) show an example of the regions used to separate arteries/arterioles and veins/venules in the three areas (color wheel velocity range: 0–20 mm/s). In (**a-2**), red is the cortex, dark blue is the outer medulla, and turquoise is the inner medulla. The yellow arrows indicate the mean angle of the arterial flow direction. (**b**) shows the separated tracks from the inserts in (**a-1**) (velocity range: 0–5 mm/s in the medulla, 0-20 mm/s in the cortex). (**c**) shows MB velocities (mean and standard deviation) from arterial vs. venous tracks (SRUS scan 1). (**d**) shows the relationship between baseline MB velocity in the arteries/arterioles of cortex and outer medulla and the MAP. AVR = ascending vasa recta, CO = cortex, DVR = descending vasa recta, IM = inner medulla, MB = microbubble, OM = outer medulla. *** = *p* ≤ 0.001, **** = *p* ≤ 0.0001. Scale bars = 2 mm.

Figure 3 illustrates how the MB velocity map segmentation allowed separation of the arterial and venous MB tracks in the three areas. By separating the descending from ascending vasa recta, the vascular organization of the medulla stood out, e.g., with a clear visualization of the descending vasa recta bundles of the outer medulla’s inner stripe (Figure 3b). In the inner medulla, the bundle organization of the descending vasa recta is lost [39], which is also evident from Figure 3b. The MB velocities in the cortical arteries/arterioles and the descending vasa recta were higher than in the cortical veins/venules and ascending vasa recta, respectively (Figure 3c, asterisks indicate results of paired *t*-test on the log-transformed MB velocities in cortical arteries/arterioles vs. cortical veins/venules (left graph, *p* = 0.0006) and square root-transformed MB velocities of the outer medulla descending vs. ascending vasa recta (right graph, *p* < 0.0001)). The ascending vasa recta are more numerous and larger in diameter than the descending vasa recta, which matches the slower velocity [40]. However, the mean number of descending vasa recta MB tracks was higher than ascending ones (SRUS scan 1: 1581 ± 366 vs. 1338 ± 398, *p* = 0.024). It could be because many MBs disrupt before reaching the ascending vasa recta, as hypothesized by Foiret et al. [25]. The baseline MB velocity in the cortical arteries/arterioles tended to decrease with decreasing MAP (Figure 3d, Pearson’s correlation of log-transformed MB velocities from the cortical arteries/arterioles and MAP: R^2^ = 0.62, *p* = 0.035.). However, some rats had a MAP below the lower limit of renal autoregulation, which usually ensures stable renal blood flow during acute changes in MAP.

### 3.2. Effects of Prazosin on MAP and Intrarenal Microbubble Velocities

**Figure 4:**
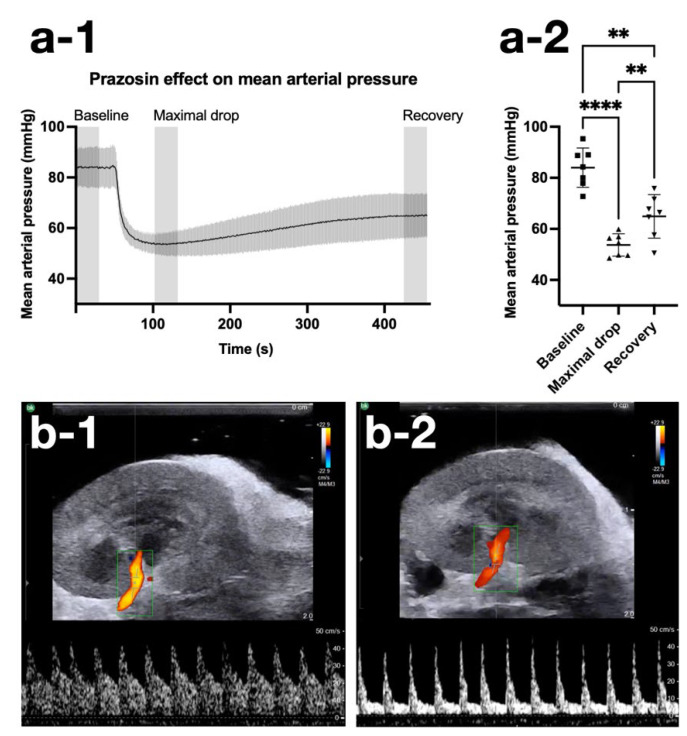
Effect of prazosin on mean arterial pressure and renal artery triplex Doppler. (**a-1**,**a-2**) show the effect of prazosin on mean arterial pressure during SRUS scan 2 (mean and standard deviation shown). (**b-1**) shows an example of the color Doppler and spectral Doppler wave pattern from the normal renal artery or one of its branches at baseline before SRUS scan 1. (**b-2**) shows a decreased color Doppler signal and altered spectral Doppler wave pattern from the same artery after completion of SRUS scan 3. ** = *p* ≤ 0.01, **** = *p* ≤ 0.0001.

The systemic delivery of prazosin was illustrated by a decrease in the MAP. The MAP was 84 ± 8 (mean ± standard deviation) mmHg during the first 30 s of SRUS scan 2 (Figure 4(a-1,a-2)), similar to MAP during SRUS scan 1 (86 ± 8 mmHg). The maximum drop in MAP occurred 52–82 s after prazosin injection (54 ± 4 mmHg). At the end of SRUS scan 2, MAP had increased to 65 ± 8 mmHg and remained low in SRUS scan 3 (65 ± 6 mmHg). The 30-s periods (grey columns in Figure 4(a-1)) were compared, showing a statistically significant decrease and increase in MAP (F(1.878, 11.27) = 65.00, *p* < 0.0001); Tukey’s multiple comparisons test is shown in Figure 4(a-2) (baseline vs. max drop *p* < 0.0001, max drop vs. recovery *p* = 0.007, baseline vs. recovery *p* = 0.002). The renal artery’s normal spectral Doppler pulsed-wave pattern with a high end-diastolic velocity (Figure 4(b-1)) changed to a pattern with low end-diastolic velocity after prazosin (Figure 4(b-2)), reflecting the decreased vascular tone. Triplex Doppler images from all rats are found in Supplementary Figure S2.

To further support the estimated MB velocities, ultrasonic perivascular flow probe measurements of renal blood flow after prazosin injection from a separate unpublished trial are found in Supplementary Figure S3. They showed that prazosin at a lower dose (0.0125 mg/kg vs. 0.1 mg/kg) significantly decreased the renal blood flow.

**Figure 5:**
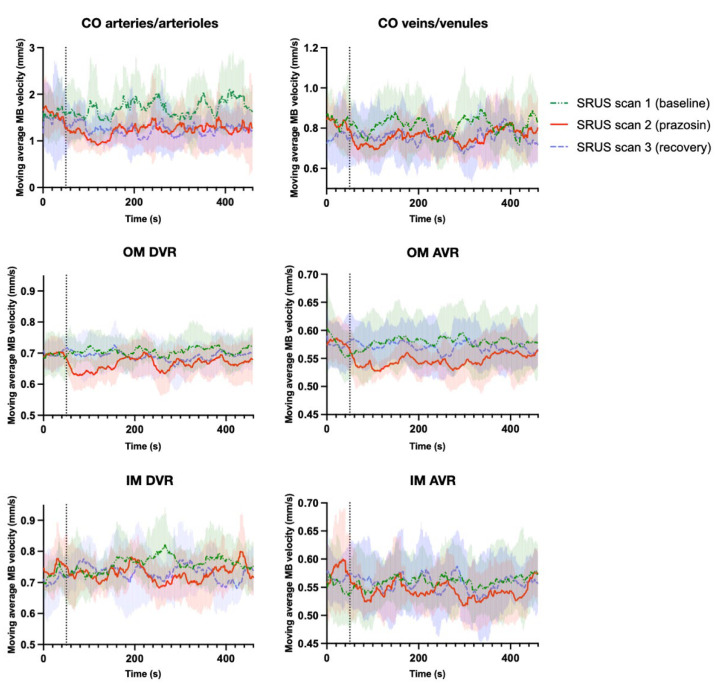
Moving average MB velocities in the arterial and venous beds of the cortex, outer medulla, and inner medulla during the three consecutive SRUS scans. The vertical dotted line indicates the time for prazosin injection. The semisolid colored areas are the standard deviations.

The moving average MB velocities from the six vascular beds during SRUS scan 1–3 are displayed in Figure 5.

In the cortical arteries/arterioles, outer medulla descending vasa recta, and outer medulla ascending vasa recta, a prazosin-induced MB velocity drop was visible from the moving average velocity graphs from SRUS scan 2 when compared with SRUS scan 1. In the cortical arteries/arterioles, the decrease seemed to continue in SRUS scan 3, while in the outer medulla ascending vasa recta and outer medulla ascending vasa recta, the velocity increased to baseline levels in SRUS scan 3. A velocity decrease in the cortical veins/venules also seemed to occur after prazosin, but similar random fluctuations were seen during SRUS scan 1. In the inner medulla descending vasa recta and inner medulla ascending vasa recta, no apparent effect of prazosin was visible.

**Figure 6:**
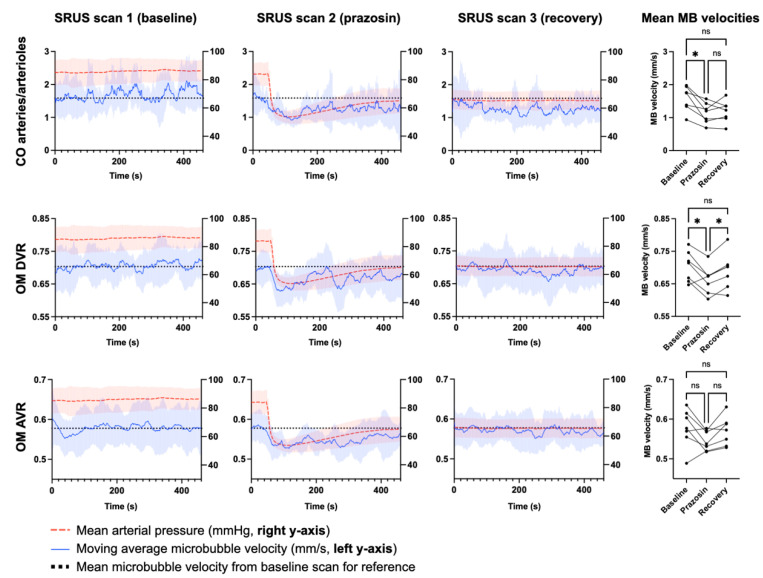
Moving average MB velocities shown together with the mean arterial pressure. Semisolid areas represent the standard deviation. The black dotted line indicates the mean MB velocity from SRUS scan 1 for an easier immediate visual comparison in SRUS scans 2 and 3. The scatter plots on the right show the mean MB velocities from the raw data of the three scans. The asterisks indicate the result of Tukey’s post-hoc test. * = *p* ≤ 0.05, ns = non-significant (*p* > 0.05).

In Figure 6, the MB velocities from the cortical arteries/arterioles, outer medulla descending vasa recta, and outer medulla ascending vasa recta are displayed along with the MAP during each scan to illustrate the temporal relationship between the two parameters.

In the cortical arteries/arterioles, prazosin lowered the MB velocities significantly (*p* = 0.013). Post-hoc comparison showed that the MB velocity decreased significantly from SRUS scan 1 to scan 2 (*p* = 0.032). Prazosin also significantly lowered the MB velocities in the outer medulla descending vasa recta (*p* = 0.026). The post-hoc comparison showed that the MB velocity decreased significantly from SRUS scan 1 to SRUS scan 2 (*p* = 0.037) and increased in SRUS scan 3 to a level significantly higher than SRUS scan 2 (*p* = 0.013). The decrease and increase in MB velocity in the outer medulla ascending vasa recta were not statistically significant (*p* = 0.064). The remaining vascular beds showed no statistically significant MB velocity alterations between scans and were not analyzed further.

The three 30-s periods within SRUS scan 2 were also analyzed for the cortical arteries/arterioles, outer medulla descending vasa recta, and outer medulla ascending vasa recta. The results were slightly different: in the cortical arteries/arterioles, the ANOVA was non-significant, but the post-hoc comparison still showed a decrease in MB velocity from the baseline period to the period with max MAP drop (*p* = 0.031). In the outer medulla descending vasa recta, all tests were non-significant. In the outer medulla ascending vasa recta, prazosin lowered the MB velocity significantly (*p* = 0.012), with the post-hoc test showing velocity decrease from baseline period to the period with max MAP drop (*p* = 0.040). The mean MB velocities and results from all ANOVA and post-hoc tests are shown in Supplementary Table S2.

The cortical arteries/arterioles have a fast flow and complex geometry. In this area there were seconds with no generated MB tracks (271 ± 46 of the 410 s of SRUS scan 1 had velocity estimations). For the outer medulla descending vasa recta with a more homogenous slow flow and a simpler vascular geometry, 405 ± 4 s had velocity estimations. Supplementary Figure S4a illustrates the difference in number of track positions/s during SRUS scan 1 in the different vascular beds. There was a steady number of counted MBs/s throughout scanning (Supplementary Figure S4b) and no correlation between MB count/s and number of seconds with velocity estimations in the cortical arteries/arterioles (Pearson’s correlation, R^2^ = 0.088, *p* = 0.519).

## 4. Discussion

In this study, manual segmentation of SRUS images was used to isolate the unique renal vascular beds of the cortex and medulla and extract MB velocities from arteries/arterioles or veins/venules separately. It was possible to detect and differentiate acute prazosin-mediated alterations in MB velocities in the separated vascular beds and display their dynamic responses using the moving average MB velocities during SRUS acquisition. An acute response to prazosin was detectable in the MB velocities from the renal outer medulla’s microvasculature, which is usually difficult to examine in vivo due to its deep location. These results support that SRUS can be used to investigate the intrarenal distribution of blood velocities in different conditions. Simultaneous quantification of the outer and inner medullary blood flow opens possibilities to investigate how regional blood flow alterations influence the corticomedullary gradients of NaCl and urea in the medulla [41,42]. Another clinically intriguing application is monitoring flow changes in the ischemia-vulnerable outer medulla [19,43].

The MB velocities decreased significantly in response to prazosin in the cortical arteries/arterioles, outer medulla descending vasa recta, and outer medulla ascending vasa recta (the latter only when analyzing the 30-s periods of SRUS scan 2). This differentiated response in MB velocity can be explained by the distribution of the α1-adrenoceptor, located on the vascular smooth muscle cells of the arteries and arterioles. Prazosin inhibits the α1-adrenoceptor causing vasodilation. When administered intravenously, prazosin reduces the total peripheral resistance and the vasodilation causes the renal blood flow and the cortical artery/arteriole MB velocity to decrease [44,45]. The descending vasa recta do not have vascular smooth muscle cells but are surrounded by pericytes. One study has shown a higher pericyte density in the outer medulla compared with inner medulla [46]. Likewise, adrenergic nerve fibers have been shown to travel along the outer medulla descending vasa recta and diminish in the inner medulla [47]. These differences could account for the difference in our findings: only in the outer medulla was a response to prazosin measured. Norepinephrine has been shown to cause outer medulla vasoconstriction in vitro and reduce medullary blood flow in vivo, supporting the presence of α1-adrenoceptors in this area [37,46,48]. However, the mentioned studies do not directly demonstrate the existence of the α1-adrenoceptor in the outer medulla descending vasa recta pericytes, and the MB velocity decrease could also be caused by dilation of the upstream efferent arterioles [49]. The outer medulla ascending vasa recta have mostly been found not associated with pericytes [50,51], but a decrease in MB velocity was found when analyzing 30-s periods of SRUS scan 2. It could be an effect of the velocity drop on the arterial side of the outer medulla circulation. Lastly, we did not find a response to prazosin in the cortical veins/venules. The α1-adrenoceptors are present in veins, but the venous basal tone is low, and prazosin may not have dilated the veins enough.

Only a few other SRUS studies have quantified MB velocity alterations in relation to pathological changes in blood flow, e.g., slower MB velocities in the brains of older compared with younger mice, or slower MB velocities in the retinal and retrobulbar vessels in the rabbit eye at high ocular pressures [27,28]. In the latter, arteries and veins were separated bi-directionally with flow towards (arteries) or away (veins) from the probe. Due to the renal vascular complexity, that approach was not feasible in our study. Similarly to our approach, a recently published study showed increased MB velocities in the renal cortical radial arteries of hypertensive rats by separating arteries and veins based on the MB flow direction towards or away from the renal surface [29]. Another recent SRUS study used the pulsatility of arteries to separate them from veins in cross-sectional images of the murine cerebral vasculature [52]. They used a high temporal resolution (1000-Hz frame rate) to capture the velocity fluctuations within a single cardiac cycle of mice with a 550-beats/min heart rate. For rats with ∼350 heart beats/min, a frame rate much higher than used in our study (54 Hz) would be required. The arterial MB tracks in the renal cortex can come from different vessel types with different velocities. In our study, some tracks were from smaller branches of the arcuate arteries, most were from cortical radial arteries, some were probably from afferent or efferent arterioles, and some MBs might even have been tracked in the capillary network (the latter two not substantiated). Depending on the type of vessel in which the MBs were tracked, estimated velocities ranged from the minimum to the maximum of our tracking algorithm’s velocity span (0–15 mm/s). Investigating them as a whole may blur the outcome of an intervention or a disease, as the vessels may respond differently. Additionally, while most of the cortical arterioles project towards the renal surface, some have a direction toward the medulla, and the same goes for the veins [53]. Accordingly, separating vessels based on direction may mix arterioles with the venules and vice versa. Moreover, capillaries run in all directions and may occur in both regions. Therefore, the separation of vessels based on pulsatility is a compelling alternative for the renal cortex [52].

The MB velocity is typically estimated as a mean of all tracks generated during data acquisition [2,3,21,25,28], but this will not necessarily reveal dynamic vascular flow alterations. Therefore, we visualized the MB velocities as moving averages during scanning and compared MB velocities from shorter 30-s intervals within the same scan. However, the data from the 30-s periods should be interpreted cautiously, as they are based on fewer velocity estimates. The estimated velocities at a given time point are highly dependent on the conditions for MB tracking, such as complexity of the vascular geometry and distribution of the MBs, variations in flow velocity and pulsatility, signal-to-noise ratio, out-of-plane motion, or errors in MB localization [32,33,54,55]. Such variations depending the on vascular complexity were illustrated with the difference in number of track positions between the intricate cortical arteries/arterioles versus the simpler outer medulla vessels (Supplementary Figure S4a). A way to optimize the regional velocity estimations would be to adjust the MB infusion concentration according to the area of interest; the highly perfused cortex could be imaged with an even lower MB concentration for better tracking.

There are some limitations to this study. Firstly, we measured only the velocity of MBs in the flowing blood, not the blood volume flow, which would require precise estimations of vessel diameters. Vessel diameters are highly dynamic [56,57], and the diameters estimated from the accumulated MB tracks do not necessarily represent a vessel diameter at a given time. As the velocity estimations from shorter periods are based on fewer tracks that most likely do not fill the vessel lumen, valid diameters cannot be extracted. The evaluation of tissue perfusion based on velocities alone should be done carefully, as higher velocities do not necessarily mean better blood flow, it also depends on the cross-sectional vessel area and number of perfused microvessels. Another limitation is the lack of a gold standard reference for verifying the MB velocities. The velocities derived from 2D SRUS are likely to be underestimated due to the out-of-plane vessels [14,33,58]. With a low frame rate, one could also speculate that only the slowest moving MBs in the periphery of a vessel with a parabolic flow are tracked. A frame rate in the kHz range would improve tracking by increasing the number of detected MBs/s and allow estimation of more realistic velocities, as exemplified in a rat brain with velocities ranging from mm/s to cm/s [2]. For dynamic evaluations of the vasculature, ultrafast Doppler or fast synthetic aperture vector flow imaging could be an alternative to SRUS [59,60]. However, these techniques still have lower spatial resolution than SRUS and Doppler will not allow, e.g., separation of the descending and ascending vasa recta in an entire cross-section of the kidney. Finally, because we used a clinical ultrasound scanner with a low frame rate, a high prazosin dose was used to ensure a measurable response in the intrarenal vessels, especially the medullary microcirculation with a slow flow. For detection of subtler velocity alterations, optimization of SRUS acquisition or post-processing parameters such as increased frame rate or improved tracking algorithms that can cope with overlapping MBs are necessary [61], especially since transcutaneous scanning needed for longitudinal studies will introduce additional challenges for MB tracking [62].

## 5. Conclusions

In conclusion, super-resolution ultrasound imaging using microbubble localization and tracking makes it possible to evaluate microbubble velocities in separated arterial and venous vascular beds of rats’ renal cortex, outer medulla, and inner medulla. In particular, the medullary microcirculation has previously been inaccessible for in vivo measurements. Therefore, super-resolution ultrasound imaging has a promising potential as a tool to investigate the intrarenal distribution of the renal blood flow velocities under different physiological and pathological conditions.

## 6. Patents

Patent on the tissue motion correction algorithm by J.A.J. and I.T. used in this study has been purchased by BK Medical ApS, Herlev, Denmark.

## Data Availability

Raw data and image processing algorithms can be exchanged through a collaboration agreement. Processed data and analysis algorithms can be made available upon request.

## Supplementary Materials

The following supporting information can be downloaded at: https://www.mdpi.com/article/10.3390/diagnostics12051111/s1, Table S1: Animal data.; Table S2: Microbubble velocities; Figure S1: Super-resolution ultrasound imaging post-processing overview. Figure S2: Triplex Doppler images; Figure S3: Prazosin effect on renal blood flow; Figure S3: Track positions and microbubble count.
