# Supplementary material for: Super-Resolution Ultrasound Imaging Can Quantify Alterations in Microbubble Velocities in the Renal Vasculature of Rats
Source: Diagnostics (Basel). 2022 Apr 28;12(5):1111. doi: 10.3390/diagnostics12051111 (PMC9140053; doi:10.3390/diagnostics12051111)
Supplement: Supplementary file 1 [file diagnostics-12-01111-s001.zip › diagnostics-1627482-supplementary.pdf]

Supplementary material

Super-Resolution Ultrasound Imaging Can Quantify Alterations in Microbubble Velocities in the Renal Vasculature of Rats

Sofie Bech Andersen[1,2,*], Iman Taghavi[3], Stinne Byrholdt Søgaard[1,2], Carlos Armando Villagómez Hoyos[4], Michael Bachmann Nielsen[2,5], Jørgen Arendt Jensen[3], Charlotte Mehlin Sørensen[1]

1 Department of Biomedical Sciences, University of Copenhagen, 2200 Copenhagen, Denmark
2 Department of Diagnostic Radiology, University Hospital Rigshospitalet, 2100 Copenhagen, Denmark
3 Center for Fast Ultrasound Imaging, Department of Health Technology, Technical University of Denmark, 2800 Lyngby, Denmark
4 BK Medical ApS, 2730 Herlev, Denmark
5 Department of Clinical Medicine, University of Copenhagen, 2200 Copenhagen, Denmark
* Correspondence: sofiebechandersen@gmail.com (S.B.A)

Supplementary Tables

Table S1. Animal data.

Rat id	Weight (g)	Mean MAP (mmHg)*			Mean MAP SRUS scan 2 (mmHg)**		
		SRUS scan 1	SRUS scan 2*	SRUS scan 3	Baseline	Max drop	Recovery
Rat 1	302	95	53	62	89	50	58
Rat 2 (excluded)	301	61	-	-	-	-	-
Rat 3	329	86	66	65	84	60	68
Rat 4	335	80	65	71	78	56	72
Rat 5	296	91	62	67	89	55	66
Rat 6	296	74	49	54	73	50	51
Rat 7	393	94	68	74	95	57	76
Rat 8	402	82	57	65	80	49	65
Mean	*332*	*86*	*60*	*65*	*84*	*54*	*65*
SD	*43*	*8*	*7*	*6*	*8*	*4*	*8*
Median	*316*	*86*	*62*	*65*	*85*	*56*	*66*
IQR	*82*	*11*	*10*	*6*	*10*	*7*	*8*

*The MAPs are a mean from the entire scan period (SRUS scan 2 after prazosin was administered). **The MAPs are a mean from 30-s periods of SRUS scan 2. IQR = interquartile range, MAP = mean arterial pressure, SD = standard deviation, SRUS = super-resolution ultrasound.

Table S2. Microbubble velocities.

	MB velocity mean (SD) in mm/s			ANOVA*		Tukey's test*		
						1 vs 2	1 vs 3	2 vs 3
**SRUS scan 1, 2, and 3 comparison**	SRUS scan 1	SRUS scan 2	SRUS scan 3	F (DFn, DFd)	P value	P values		
CO arteries/arterioles	1.59 (0.38)	1.14 (0.31)	1.18 (0.33)	F (1.796, 10.78) = 6.902	***0.0133***	***0.0318***	0.0626	0.9603
CO veins/venules	0.79 (0.09)	0.74 (0.08)	0.74 (0.10)	F (1.870, 11.22) = 2.702	0.1124	0.3019	0.1557	0.6902
OM DVR	0.70 (0.05)	0.66 (0.04)	0.69 (0.06)	F (1.347, 8.080) = 6.691	***0.0261***	***0.0371***	0.6306	***0.0132***
OM AVR	0.58 (0.05)	0.55 (0.03)	0.57 (0.04)	F (1.417, 8.502) = 4.197	0.0635	0.1536	0.7711	0.0611
IM DVR	0.75 (0.05)	0.72 (0.03)	0.73 (0.07)	F (1.637, 9.823) = 1.570	0.2529	0.2254	0.3063	0.9914
IM AVR	0.55 (0.05)	0.54 (0.04)	0.56 (0.05)	F (1.776, 10.66) = 1.857	0.2042	0.2778	0.9987	0.1992
**SRUS scan 2 comparison**	Baseline	Max drop	Recovery	F (DFn, DFd)	P value	P values		
CO arteries/arterioles	1.59 (0.53)	0.92 (0.19)	1.28 (0.75)	F (1.281, 7.688) = 2.257	0.1734	***0.0314***	0.4878	0.8873
OM DVR	0.70 (0.05)	0.64 (0.04)	0.67 (0.06)	F (1.893, 11.36) = 2.488	0.1287	0.1785	0.4356	0.5807
OM AVR	0.58 (0.04)	0.53 (0.02)	0.55 (0.03)	F (1.520, 9.119) = 8.188	***0.0123***	***0.0401***	0.1144	0.1253

*ANOVA and Tukey's test were done on log-transformed data for the cortex and square root-transformed data for the medulla. 1 vs. 2, 2 vs. 3, and 1 vs. 3 refer to the three SRUS scans. AVR = ascending vasa recta, CO = cortex, DVR = descending vasa recta, IM = inner medulla, MB = microbubble, OM = outer medulla.

Supplementary Figures

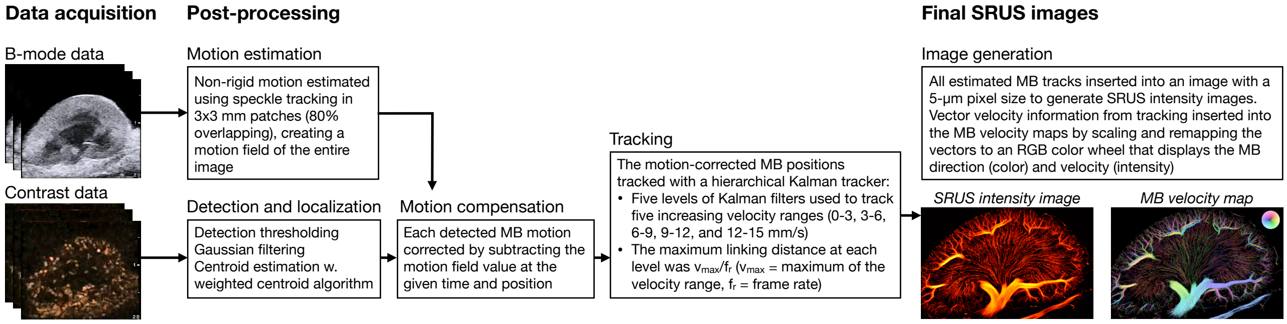

Figure S1. Super-resolution ultrasound imaging post-processing overview. MB = microbubble, SRUS = super-resolution ultrasound.

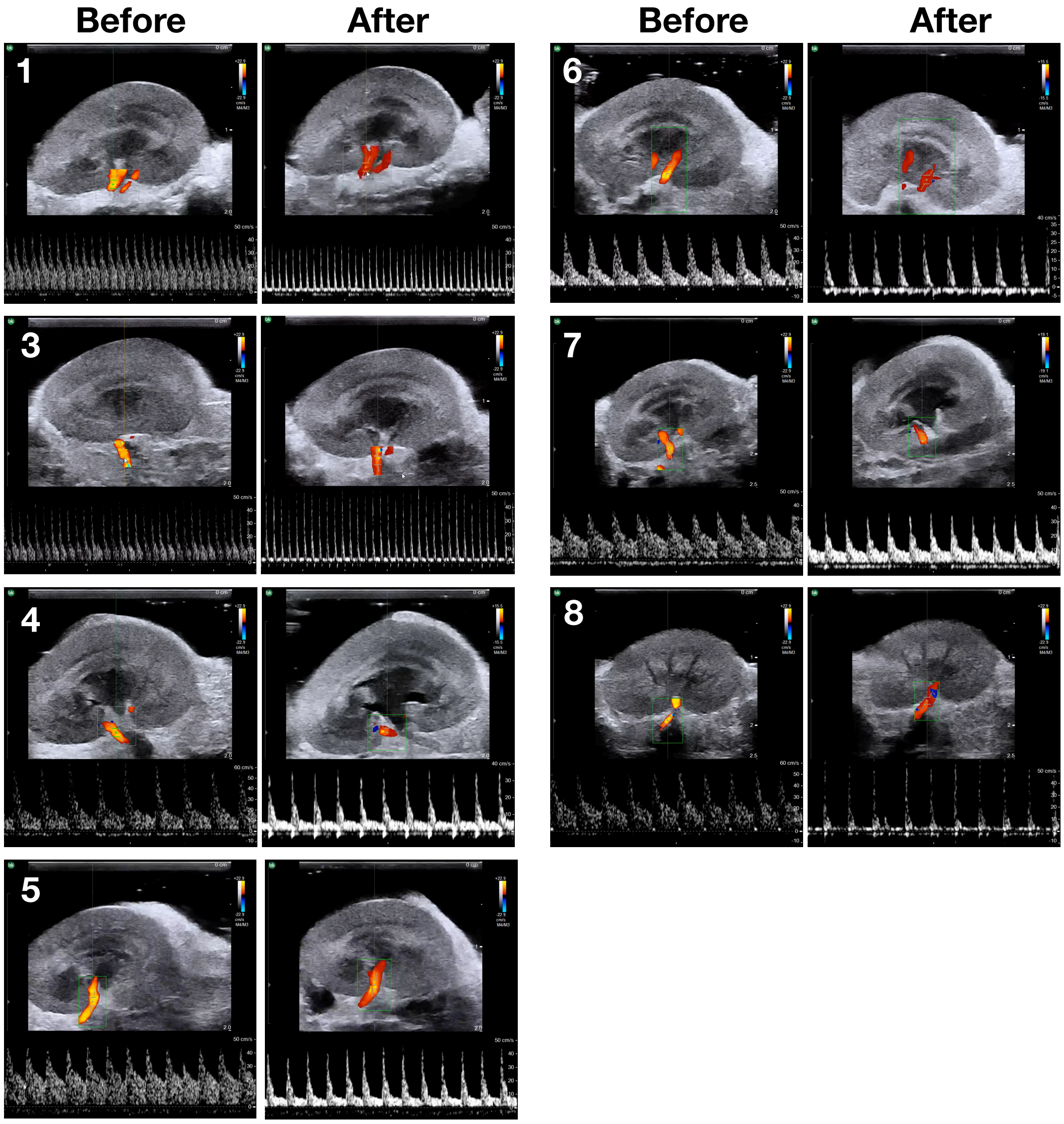

Figure S2. Triplex Doppler images. Shows triplex Doppler measurements on the renal artery or one of its branches outside or at the renal hilum before SRUS scan 1 and after SRUS scan 3. They show a reduced color Doppler intensity combined with reduced diastolic flow velocity after SRUS scan 3 in response to prazosin. Note that the shown time interval for the spectral Doppler for rat 1 and 3 is longer than for the remaining rats. The white number in upper left corner is animal id.

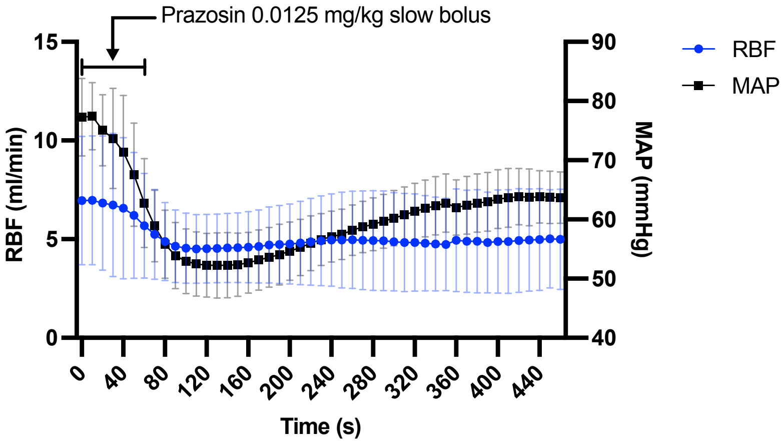

Figure S3. Prazosin effect on renal blood flow. Shows measurements of MAP and RBF (measured with an ultrasonic perivascular flow probe around the renal artery (Transonic Systems Inc., Ithaca, NY, USA)) in response to a slow bolus of 0.0125 mg/kg prazosin. The data are from another unpublished experiment with five male Sprague Dawley rats. The RBF decreased significantly from 7.2±3.2 ml/min in the first 30 sec (MAP = 76±5 mmHg) to 4.8±1.8 ml/min during the 30 sec with max drop in MAP (MAP = 51±5 mmHg) (p = 0.034). MAP = mean arterial pressure, RBF = renal blood flow.

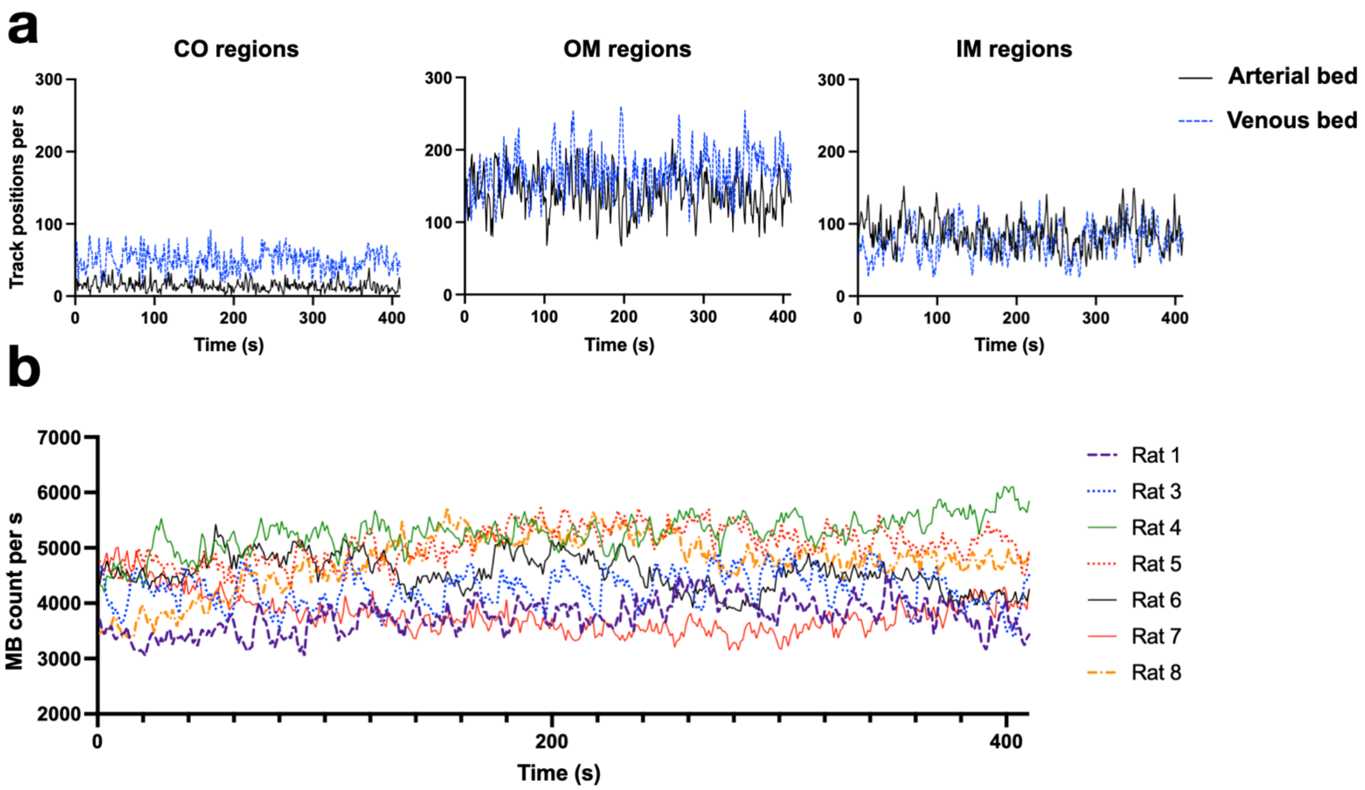

Figure S4. Track positions and microbubble count. **(a)** Shows the total number of track positions per sec for SRUS scan 1 in cortex, outer, and inner medulla. The track positions refer to the number of positions (MBs) that were used to generate velocity estimations. The graphs illustrate how there are more MBs to track in the straight vasa recta with slow flow compared with the cortex which has a more complex geometry, is denser, receives more blood and hence more MBs than the medulla, and contains a mixture of vessel types with both slow and fast flow (minding that the cortical area is smaller than outer and inner medulla (23.7±3.1 vs. 43.6±6.4 and 27.4±7.0 mm^2, respectively)). **(b)** Shows the total MB count per sec in the whole kidney in each rat during SRUS scan 1. CO = cortex, IM = inner medulla, OM = outer medulla.
